# Edible Insects and Allergenic Potential: An Observational Study About In Vitro IgE-Reactivity to Recombinant Pan-Allergens of the Black Soldier Fly (*Hermetia illucens*) in Patients Sensitized to Crustaceans and Mites

**DOI:** 10.3390/ijms262210831

**Published:** 2025-11-07

**Authors:** Francesca Nicoletta, Erminia Ridolo, Martina Ottoni, Alessandro Barone, Danila Delfino, Claudia Folli, Tullia Tedeschi

**Affiliations:** 1 Departmental Unit of Allergology, Guglielmo da Saliceto Hospital, 29121 Piacenza, Italy; f.nicoletta@ausl.pc.it; 2 Department of Internal Medicine, University of Parma, 43121 Parma, Italy; 3 Allergy and Clinical Immunology, Medicine and Surgery Department, University of Parma, 43121 Parma, Italy; martina.ottoni@unipr.it (M.O.); alessandro.barone@unipr.it (A.B.); 4 Department of Food and Drug, University of Parma, 43121 Parma, Italy; danila.delfino@unipr.it (D.D.); claudia.folli@unipr.it (C.F.); tullia.tedeschi@unipr.it (T.T.)

**Keywords:** arginine kinase, black soldier fly, crustaceans, edible insects, food allergy, *Hermetia illucens*, novel foods, tropomyosin

## Abstract

Given their nutritional features and environmental sustainability, black soldier fly (*Hermetia illucens*) larvae are currently being considered in Europe for commercialization as human food. The primary goal of this study is the determination of in vitro IgE-cross-reactivity to recombinant tropomyosin (_HI_TPM) and arginine kinase (_HI_AK) of *H. illucens* in subjects sensitized to crustaceans and/or mites. Dot blot assays for recombinant _HI_TPM were carried out with the sera of 48 subjects, 30 sensitized to crustaceans (Cr+) and/or house dust mites (HDM+) (STUDY group) and 18 non-sensitized (CTRL group). A higher rate of IgE-reactivity to recombinant _HI_TPM was found in the STUDY group compared to non-sensitized controls (73% vs. 44%; *p* 0.066). No significant relationship was achieved upon dot blot assays for _HI_AK. No relevant association between a positive history of food reactions and immunoreactivity to _HI_TPM and to _HI_AK was reported (15% in _HI_TPM+ vs. 6% in _HI_TPM-, *p* NS; 28% in _HI_AK+ vs. 50% in _HI_AK-, *p* NS), contrary to the _HI_TPM+Cr+HDM+ subset (50% vs. 0%, *p* 0.022). Considering the wide overlap of pan-allergens within the *Arthropoda* phylum, concerns about allergenic potential due to the eventual consumption of *H. illucens*-enriched foods might be valid. Therefore, targeted studies involving basophil activation tests, skin prick tests, and a double-blind placebo-controlled oral food challenge using *H. illucens* are needed.

## 1. Introduction

According to the Food and Agriculture Organization, the global population will reach 9.1 billion in the next 25 years [1]. Concurrently, inexorable climate change undermines the stability of global food systems, exposing populations, especially those who are socio-economically vulnerable, to different forms of malnutrition [2]. As a consequence, the world’s overcrowding and the global warming highlight the need to find new edible resources as alternatives to traditional animal proteins. This necessity has led to ever-growing attention to a particular category of food sources, labelled “novel foods”. Novel foods are defined as foods not consumed to a significant degree by humans in the European Union before 15 May 1997 (the date of the first Regulation on this subject). They include foods newly developed using advanced technologies and production processes, as well as food which is, or has been, traditionally eaten outside of the European Union [3].

In such a scenario, edible insects may represent ideal candidates as a sustainable nutritional resource thanks to their amount of polyunsaturated fatty acids, essential amino acids, proteins, and trace elements. In addition, compared to traditional livestock farming, insect breeding is related to lesser greenhouse gas production, water pollution, and impact on the soil, as well as greater food conversion efficiency [4].

On the basis of the positive opinions expressed by the European Food Safety Authority (EFSA), the European Commission previously authorized the commercialisation of four species of insects as human edible sources: the yellow mealworm larva (*Tenebrio molitor*, in frozen, dried and powder formulations), the lesser mealworm (*Alphitobius diaperinus*, in frozen and freeze-dried formulations), the locust (*Locusta migratoria*, in frozen, dried, and powder formulations), and the domestic cricket (*Acheta domesticus*, in frozen, dried, powder, and partially defatted formulations) [5].

Although their advantages may be considerable, it is also true that edible insects are characterized by a poor history in the cuisine of Western countries, and therefore they represent a relatively unknown food. For this reason, in recent years, insects have attracted much interest in the scientific community, particularly regarding their chemical, biological and allergological safety. Several cases of allergic reactions after the ingestion of insects have been reported in the literature, with a spectrum of symptoms extending from mild local reactions to anaphylactic shock [6]. The causes of these reactions may be attributable to genuine sensitizations for specific insects (particularly in Asiatic and African regions, where entomophagy is a common practice) but also to cross-reactions due to sensitizations to other species sharing phylogenetically preserved allergens [7]. Among the well-known pan-allergens of insects, tropomyosin (TPM) and arginine kinase (AK) are the most important, causing cross-reactivity with the homologous proteins present in more widespread foods (i.e., seafood), food parasites (i.e., *Anisakis simplex*), and inhalant agents (i.e., mites and cockroaches) [7]. Moreover, in case of reaction to edible insects, lesser-known pan-allergens shared by invertebrates should also be considered, such as alpha-actin, enolase, fructose 1,6-biphosphate aldolase, glyceraldehyde-3-phosphate dehydrogenase, apolipophorin III, larval cuticle protein, and activated protein kinase receptor [7,8]. Allergens belonging to the families of chemosensorial proteins, hexamerins, and odorant-binding proteins instead were found to be more specific for edible insects [8].

Within this general framework, other species of insects currently represent valuable candidates to join the group of those already approved for commercialization in the European Union as human food [9]. Due to their ability to convert a wide range of organic matter in body mass, including human faeces, abattoir waste, and organic waste from crop agriculture, the larvae of the black soldier fly (*Hermetia illucens*) have already demonstrated their potential in the context of animal feed. Thanks to their elevated content of proteins, fat (30–53 g/ and 20–41 g/100 g of dry matter, respectively), and micronutrients (especially calcium, iron and zinc), *H. illucens* larvae might represent a valuable source of food nutritional enrichment [10].

From an allergological perspective, TPM, AK, and other pan-allergens—such as myosin light chain, troponin T, glyceraldehyde 3-phosphate dehydrogenase and triosephosphate isomerase—were identified in the extract of *H. illucens* using liquid chromatography–mass spectrometry analysis [11]. Interestingly, a shotgun proteomic approach combined with in silico assessment revealed TPM as the major pan-allergen of the *H. illucens* proteome, with a strong similarity with TPM of crustaceans and mites [12]. In agreement with these studies, our previous studies led to the characterization of peptide sequences related to TPM and AK of *H. illucens* by combining bioinformatic analysis and mass spectrometry approaches. The recognition of pan-allergens by IgE from sera of patients allergic to crustaceans and mites was demonstrated using immunoblotting assays [13,14]. Moreover, another study confirmed the cross-reactivity of shrimp-specific antibodies to TPM of *H. illucens* and identified additional unique allergens of the black soldier fly, including hemocyanin, vitellogenin, heat shock protein 20, apolipophorin-III, and chitin-binding protein [15].

Starting from these premises, it is reasonable to speculate that patients allergic to crustaceans and/or mites might be more likely to experience allergic reactions after the eventual ingestion of *H. illucens*. On the other hand, it is known that an eventual cross-reactivity does not necessarily imply the onset of allergic manifestations after the consumption of insects. The aim of the study, therefore, was primarily to determine whether sera from non-insect consumers sensitized to crustaceans and/or mites cross-react with recombinant pan-allergens of *H. illucens* (tropomyosin—_HI_TPM, and arginine kinase—_HI_AK) in vitro. Furthermore, the secondary objective was to assess whether the patients whose sera reacted to _HI_TPM and _HI_AK reported a higher history of allergic reactions to food both in the entire cohort and in subsets that differ in their profile of sensitization to house dust mites (HDM) and crustaceans.

## 2. Results

### 2.1. Characteristics of the Cohort

The starting cohort involved 50 subjects. Based on their sensitization profile, subjects were assigned to the study group (named “STUDY”, including patients sensitized to crustaceans and/or HDM) or to the control group (named “CTRL”, non-sensitized). Within the STUDY group, 11 patients were found to be sensitized only to *Dermatophagoydes pteronyssimus* and/or *Dermatophagoydes farinae* (HDM+) and 19 to both crustaceans and HDM (Cr+ HDM+).

**Figure 1:**
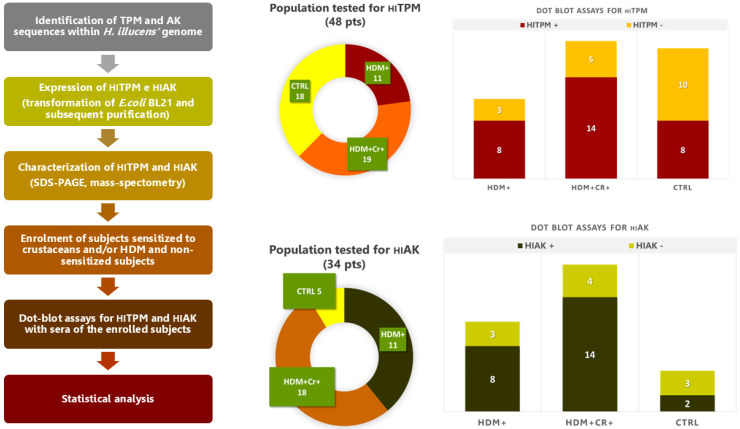
Summary of all the phases leading to the dot blot assays with sera of the enrolled subjects for the recombinant pan-allergens of *H. illucens* (_HI_TPM and _HI_AK) and respective results. Abbreviations: Cr—crustaceans, CTRL—control study, HDM—house dust mite, _HI_AK—arginine kinase of *H. illucens*, _HI_TPM—tropomyosin of *H. illucens*. The results of dot blot assays were overall categorized as “_HI_TPM+” only in the case of positive outcomes for both recombinant variants.

**Figure 2:**
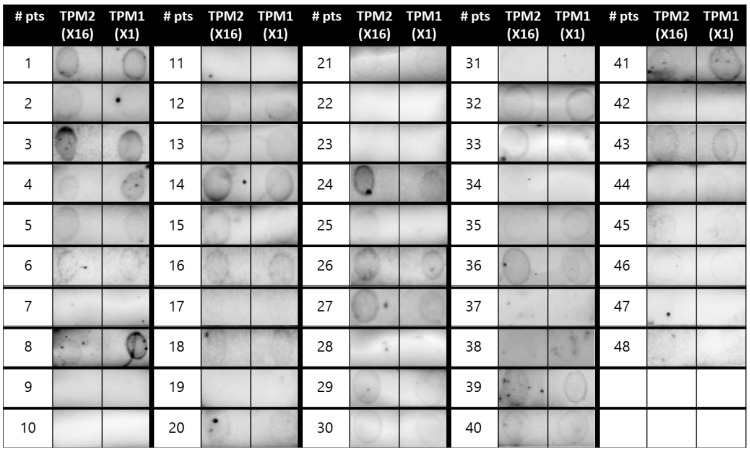
Dot blot assays for the two variants of recombinant tropomyosin of *H. illucens* _HI_TPM1-X1 and _HI_TPM2-X16.

Figure 1 summarizes the process leading to dot blot assays for the recombinant pan-allergens of *H. illucens* and the respective results. All enrolled subjects underwent blood sampling for sera extraction, which was needed to carry out dot blot assays with the recombinant proteins of *H. illucens*. The sera of two patients were discarded (one because of excessive lipidic components and one for contamination). Dot blot assays for two different recombinant variants of _HI_TPM (_HI_TPM1-X1 and _HI_TPM2-X16; Figure 2) were carried out for all 48 remaining patients (30 from the STUDY group and 18 from the CTRL group; Figure 1). Due to insufficient samples, the subsequent dot blot assays for a recombinant variant of _HI_AK were carried out only for 34 patients (29 STUDY and 5 CTRL; Figure 1).

The specific characteristics of the enrolled cohort, categorized as the STUDY group and CTRL group, are reported on Table 1. Some 40% of the STUDY group and 50% of the CTRL group were male. The mean age of the STUDY group was lower compared to the CTRL group (41.6 vs. 54.1 years old). The majority of the STUDY group (63%) was sensitized both to crustaceans and HDM, whereas 37% were sensitized only to HDM. None of the enrolled subjects were sensitized solely to crustaceans.

### 2.2. IgE-Reactivity to Recombinant _HI_TPM and _HI_AK Is Higher in the STUDY Group

**Figure 3:**
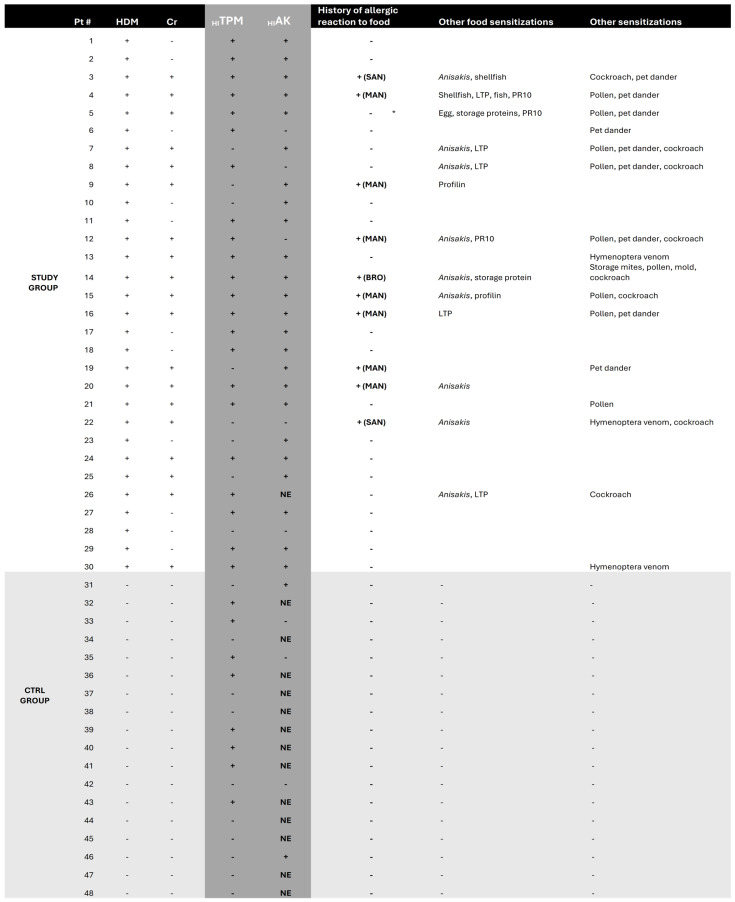
Results of skin prick tests for house dust mites and crustaceans and of dot blot assays for _HI_TPM and _HI_AK. Further information is reported, such as history of allergic reaction to any food and profile of sensitizations for each enrolled subject. Abbreviations: BRO—bronchospasm, Cr—crustaceans, HDM—house dust mites, _HI_AK—arginine kinase of *H. illucens*, _HI_TPM—tropomyosin of *H. illucens*, LTP—lipid transfer protein, MAN—mild anaphylaxis, NE—not executed, PR10—pathogen-related protein 10, SAN—severe anaphylaxis. * subject #5 never experienced a food reaction more severe than eczema.

Regarding the _HI_TPM1-X1 recombinant variant, 73% of the STUDY group were positive upon dot blot assays, while the CTRL group reported 44% positivity (*p* 0.066). Dot blot assays for the _HI_TPM2-X16 variant were positive for 77% of the STUDY group and 44% of the CTRL group (*p* 0.032). Only patient #25 from the STUDY group reported discordant results for dot blots performed for the two _HI_TPM variants, which were negative for _HI_TPM1-X1 and positive for _HI_TPM2-X16 (Figure 2). Since these two investigated variants represent the two different loci encoding for _HI_TPM in the genome of *H. illucens*, the results of dot blot assays were overall categorized as “_HI_TPM+” only in the case of positive outcomes for both recombinant variants, in order to secure the representation of both of them (73% in STUDY group vs 44% in CTRL group; *p* 0.066). Discordant or double negative results were considered “_HI_TPM−” (Figure 1 and Figure 3).

Multiple logistic regression rejected the association between sensitization to crustaceans and/or HDM and age with _HI_TPM+. Notably, the odds of _HI_TPM+ were over three times higher for subjects in the STUDY group compared to the reference group, but no statistical significance was achieved (OR 3.03, 95% CI: 0.80–11.54; *p* NS). Age was not a significant predictor (OR 1.01, 95% CI: 0.97–1.06; *p* NS). Comparable results were found considering only the outcomes for _HI_TPM2-X16 for the variables “sensitization to crustaceans and/or HDM” (OR 3.32, 95% CI: 0.87–12.68; *p* 0.079) and “age” (OR 1.01, 95% CI: 0.97–1.05; *p* NS).

In order to better characterize the STUDY cohort, the subsets HDM+Cr− and HDM+Cr+ were alternatively compared to the CTRL group, but in these cases, no significant association with positive IgE-reaction to _HI_TPM was highlighted.

Within the entire STUDY population, only 10 subjects were previously tested for *Anisakis simplex*; despite a higher rate of IgE-reactivity to _HI_TPM in patients sensitized to *A. simplex* (Anisakis+) compared to non-sensitized (70% vs. 10%, respectively), no significant correlation was described, probably due to the limited subset of patients assessed for this parameter.

Dot blot assays for _HI_AK were much more positive in the STUDY group compared to the CTRL one (83% vs. 40%), but no statistical relevance was found with the STUDY group (OR 8.04, 95% CI: 0.83–78.31, *p* 0.073) or with the specific subsets of patients HDM+Cr− (*p* NS), HDM+Cr+ (*p* NS), and HDM+Cr+Anisakis+ (*p* NS).

### 2.3. HDM+ Cr+ _HI_TPM+ Patients Reported a Higher Rate of Allergic Reactions to Food

Considering the parameter “IgE-reactivity to _HI_TPM”, no relationship with previous experiences of allergic reactions to any food was found (15% of the _HI_TPM+ subset versus 6% of the _HI_TPM− subset, *p* NS); the same was true for the “IgE-reactivity to _HI_AK” parameter (28% of the _HI_AK+ subset and 50% of the _HI_AK- subset, *p* NS).

Overall, _HI_TPM+ subsets of the STUDY and CTRL groups showed no relevant differences regarding the rate of previous allergic reactions to food (32% vs. 0%, *p* NS) (Table 2). In particular, none of the patients in the HDM+Cr−_HI_TPM+ subset ever experienced episodes of food allergy. The HDM+Cr+_HI_TPM+ subset instead reported a significant higher history of allergic reactions to food when compared to the CTRL _HI_TPM+ subset (50% vs. 0%, *p* 0.022) (Table 2). Despite a similar rate, no significant association was found for the HDM+Cr+_HI_AK+ subset (50% vs. 0%; *p* NS) (Table 2), probably due to the limited number of dot blot assays performed for _HI_AK in the CTRL group (only five) (Figure 1 and Figure 3).

## 3. Discussion

Contrary to the well-characterized allergies to crustaceans and HDM, the fragmentary information regarding allergic reactions exacerbated by entomophagy mostly originates from Asia, where this practice is widely accepted compared to the Western countries [7]. Narrowing it down to allergy to *H. illucens*, the literature is even poorer, since other insects are more commonly commercialized and worldwide consumed than flies (accounting only for 2%), i.e., beetles (31%), caterpillars (18%), *Hymenoptera* (bees, wasps, ants, at 14%), and *Orthoptera* (locusts and crickets, at 13%) [16,17].

HDM allergy is estimated to affect 1–2% of the global population [18], while allergy to crustaceans is estimated to involve up to 4% of the population in particular regions of the world [19]. On the other hand, due to the worldwide differences regarding entomophagy, there is no clear estimate about the relevance of insect food allergy. A cross-sectional analysis of medical records from a tertiary care hospital in Thailand explored the causes of 439 cases of anaphylaxis, reporting fried insects as responsible for 7.5% and ranking them as the second most prevalent food trigger in the children, after shrimps [20]. A recent Italian study instead assessed the rate of IgE-reactivity (7.5%) for any one of the three insects *Acheta domesticus*, *Locusta migratoria*, and *Tenebrio molitor* by performing the Allergy Explorer-ALEX-2 test on the sera of 2014 subjects. Tropomyosin of these insects had positive results in 34% of the cases, and arginine kinases was positive in 18.5% [21].

To our knowledge, our study is one of the few focusing on the allergenic potential of *H. illucens*, and it is the first to investigate the rate of in vitro IgE-reactivity to _HI_TPM and _HI_AK in patients sensitized to crustaceans and/or mites compared to non-sensitized controls. Our study highlighted a relevant rate of dot blot positivity for _HI_TPM (73%) in patients sensitized to crustaceans and/or HDM, and this is in agreement with the 73% of sensitization to *H. illucens* assessed by Broekman et al. in a crustacean-sensitized cohort of 15 subjects whose sera were used to perform dot blot assays with extract of black soldier fly [11]. On the contrary, no similar association was found in our study for _HI_AK. It is necessary to underline, though, that one of the limitations of our study was the very restricted number of controls assessed for _HI_AK (only five subjects) because of an insufficient volume of serological samples. Moreover, the non-transcurable rate of positivity for _HI_TPM and _HI_AK in the CTRL group (44% and 40%, respectively) might suggest a certain likelihood of achieving false positivity for the assays performed. In relation to this, it is necessary to remember that _HI_TPM and _HI_AK, used to perform dot blot assays, were not directly isolated from *H. illucens*, but they were the result of bioinformatic identification and biochemical reconstruction, which might not reflect actual real-life reactivity to *H. illucens*. In addition, since all participants were non-insect consumers, reports of positive IgE-reactivity to _HI_TPM and _HI_AK are mostly assumed to be a result of cross-reactivity to the homologous pan-allergens of crustaceans and HDM. However, it is equally true that eventual previous exposure to inhalation, physical contact, or accidental ingestion of *H. illucens* cannot be excluded.

In light of the wide overlap of TPM and AK within the *Arthropoda* phylum, and in consideration of the high prevalence of allergies to crustaceans and HDM all over the world [18,19], certain concerns regarding the allergenic potential of consumption of *H. illucens*-enriched foods are understandable. Our results highlighted a trend in IgE of patients sensitized to crustaceans and/or mites, which might be more likely to recognize homologous proteins of insects (in this case, _HI_TPM) compared to the IgE of non-sensitized patients, thus potentially suggesting immunological cross-reactivity. For clarity, no statistical relevance was found by logistic regression, but the odds of _HI_TPM positivity upon dot blot assay were over three times higher for subjects in the STUDY group compared to the CTRL group, particularly for the _HI_TPM2-X16 variant, which also achieved statistical relevance in the contingency table (*p* 0.032). However, the detection of IgE capable of binding an allergen is not enough to guarantee the onset of clinical allergic manifestations; thus, the detection of in vitro cross-reactivity does not necessarily imply greater susceptibility to allergic reactions. In this respect, another limitation of the study was the impossibility of performing additional tests that aim to better define the effective allergenicity of *H. illucens*, such as the basophil activation test (BAT), skin prick tests (SPTs), and the double-blind placebo-controlled oral food challenge test (DBPCOFC). That said, the history of previous allergic reactions to any food (sharing pan-allergens or potentially parasitized) was investigated as a surrogate parameter, and no relevant association with the state of immunoreactivity to _HI_TPM and _HI_AK was found in the overall STUDY group. On the contrary, the HDM+Cr+_HI_TPM+ subset only reported a significantly higher history of food allergic manifestations compared to the CTRL _HI_TPM+ subset, thus suggesting that an eventual mono-sensitization to *H. illucens* does not expose patients to risk of food reactions such as a triple sensitization to *H. illucens*, crustaceans and HDM. Regarding positive IgE-reactivity to other edible insects (*Acheta domesticus* and *Tenebrio molitor*), Scala et al. suggested a weaker role of co-sensitization to mites compared to co-sensitization to crustaceans [21].

It is true, however, that useful data might be obtained by real-life studies performed on patients who already experienced the consumption of *H. illucens* larvae as protein source. In this regard, a Thai survey conducted on 140 subjects estimated 12.9% of self-reported allergic reactions after entomophagy and identified a cluster of seafood-allergic patients as more prone to experiencing hypersensitivity reactions. The most often indicated as culprits were silkworm larva, grasshoppers, crickets, and bamboo caterpillars (44%, 22%, 16%, and 16%, respectively). No cases were attributed to *H. illucens*, and it is not clear if this was due to an actual lower allergenic potential or purely to minor consumption [6].

In view of all this, it is evident that allergological knowledge about the human consumption of edible insects in general, and about *H. illucens* in particular, is still in its infancy, and much has yet to be assessed in view of the eventual commercialization of the black soldier fly. Moreover, further areas for more in-depth research are physical, chemical and biomolecular processing methods, which could alter the structure of allergens and therefore the allergenic risk related to this insect source [22].

## 4. Materials and Methods

### 4.1. Enrollment of the Population

This research was carried out thanks to the collaboration between the Allergology and Clinical Immunology Service of the University Hospital of Parma and the Department of Food and Drugs of the University of Parma. The Allergology and Clinical Immunology Service was involved in the evaluation of the allergic history of the enrolled subjects, the performance of SPTs for crustaceans and HDM, and the sampling of sera used to assess immunoreactivity towards recombinant _HI_TPM and _HI_AK. The Department of Food and Drugs dealt with the preliminary process of identification and production of the recombinant variants of _HI_TPM and _HI_AK and with the dot blot assays performed on sera of the enrolled cohort to immunodetect IgE linking the pan-allergens.

The protocol of the study achieved approval (N° 19448) from the Ethical Committee of the University of Parma. Informed consent was signed by all enrolled subjects.

Inclusion criteria for the study were (1) age > 18 years; (2) expression of signed informed consent; and (3) no concomitant biologic therapy with Omalizumab. The allergological history of the patients was investigated, with a particular focus on their history of food allergic reactions (i.e., urticaria, angioedema, swelling of the throat, bronchospasm, nausea, emesis, diarrhoea, lipothymia, and syncope) and eventual treatment in the emergency room; previous documentation about specific IgE levels was also noted. SPTs for *Dermatophagoides pteronyssimus*, *Dermatophagoides farinae*, and prawn (Lofarma, Milan, Italy) were performed for all enrolled subjects according to the international standards [23]. SPTs with histamine and physiological solution were used as positive and negative controls, respectively. Tests were deemed valid when, after 15 min of application, a wheal ≥ 3 mm diameter was achieved for histamine but not for physiological solution. SPTs for the three allergens tested were considered positive when the corresponding wheals were of the same/higher diameter as the one for histamine in the same subject.

### 4.2. Identification and Production of Recombinant Variants of _HI_TPM and _HI_AK

Starting from the tropomyosin sequence of *Drosophyla melanogaster*, the sequences encoding for _HI_TPM variants were searched on the complete genome of *H. illucens* deposited in the NCBI database (accessed on November 2021), using the tBlastn algorithm.

Two genes were identified on the chromosome 6: LOC119658701 and LOC119658706. For the LOC119658701 gene, 21 splicing variants were predicted from its transcription, with a sequence identity ranging from 52% to 92%. For the LOC119658706 gene, two distinct _HI_TPM variants were predicted, sharing 95% sequence identity.

Based on the observation that most TPM isoforms known as allergens in different species arthropods are composed of 284 amino acids, all the _HI_TPM sequences with a molecular weight higher than 45 kDa were excluded from further analysis. The remaining seven protein sequences were aligned and compared with allergenic TPM from mites, crustaceans and molluscs, highlighting high sequence identity (61–80%) with allergenic TPM from mites and crustaceans. Lower sequence identities were instead observed with TPMs of molluscs (50–60%).

Observations about the conservation of the amino acid regions recognized as epitopes in the allergens Pen a 1 and Der p 10, literature reports regarding TPM peptides identified in total protein extracts of larvae of *H. illucens*, and a search in the Sequence Read Archive containing the results of RNAseq experiments conducted on larvae of *H. illucens* adapted to different substrates led to the selection of the two best recombinant proteins for evaluating the allergenicity to *H. illucens* in patients sensitized to mites or crustaceans: _HI_TPM1-X1 (NCBI protein id XP_037922243) for the LOC119658706 gene and _HI_TPM2-X16 (NCBI protein id XP_037922235) for the LOC119658701 gene [13].

In particular, the sequence identity of _HI_TPM1-X1 was 76% for Der p10 and 79% for Pen a1. The sequence identity of _HI_TPM2-X16, instead was lower (63% and 65%, respectively). More detailed descriptions of the primary structure of the two produced recombinant proteins are reported in our previous publication [13].

A similar process was performed to obtain the encoding sequence for _HI_AK starting from the sequence of the allergenic AK of the white shrimp *Litopenaeus vannamei* by accessing the NCBI database in March 2023. Two genes were identified: LOC119647699 on chromosome 2 and LOC119659652 on chromosome 6. From their transcriptions, four different variants and one different variant were predicted, respectively. The most similar sequence investigated from *H. illucens* with allergenic homologous of other arthropods (77–88%) was the isoform _HI_AK-X4 (NCBI protein id XP_037904712). In particular, _HI_AK-X4 shares 78% of sequence identity with Der p 20 (AK of *Dermatophagoydes pteronyssimus*) and 82% with Pen m 2 (AK of *Penaeus monodon*) [14]. More details about the primary structure of the produced recombinant proteins are reported in another previous publication [14].

Recombinant proteins were purified and characterised using SDS–PAGE, size exclusion chromatography, mass spectrometry, circular dichroism, and functional assays, and these data confirm high purity and correct folding, thereby minimising the likelihood that protein quality influenced IgE binding [13,14]. cDNAs encoding for _HI_TPM and _HI_AK isoforms, optimized for the expression in *E. coli* and cloned in the pEX-A128 vector (Eurofins, Milan, Italy), were subcloned in the expression vector pET28b (Merck, Darmstadt, Germany). The plasmids pET28b-TPM1-X1, pET2b-TPM2-X16, and pET28b-AK4-X4 were used to transform *E. coli* BL21 (DE3) cells (Merck). Protein expression was induced by using isopropyl 1-thio-β-D-galactopyranoside, and the bacterial cells were lysed by sonication. The recombinant proteins were purified from the soluble fraction by affinity chromatography on a His-Trap column (GE Healthcare, Chicago, IL, USA). Aliquots of purified proteins were stored in liquid nitrogen at −80 °C.

### 4.3. Immunoblotting Assays

Immunoblotting assays (dot blot) for _HI_TPM1-X1, _HI_TPM2-X16, _HI_AK4-X4 were performed with the same protocol in different sessions: aliquots (0.8 μg) of the recombinant *H. illucens* proteins were spotted on a nitrocellulose membrane (GE Healthcare), which was allowed to dry at room temperature, and then incubated with blocking solution. Successively, IgE-immunoblotting assays were carried out by incubation (1 h at room temperature) with diluted sera (1:15) from subjects sensitized to crustaceans and/or HDM or non-sensitized. The serum dilution of 1:15 was established based on optimization performed in our previous studies, where this dilution provided the best signal-to-noise ratio. Therefore, the same dilution was applied in the current study to ensure consistency and comparability of results. After washing, a further incubation (1 h at room temperature) was performed with goat anti-human IgE antibodies labelled with DyLight 680 (Microtech, Napoli, Italy) diluted 1:1000. Dot blot images were recorded by using a ChemiDoc MP imaging system (Bio-Rad, Hercules, CA, USA) and analysed with Image Lab software 3.0.1 (Bio-Rad). Positivity in the dot blots was determined by visual scoring based on the presence of a spot signal, and the same criteria were applied consistently across all experiments. In our previous study [13], BSA was used as an internal negative control to assess signal specificity. In the current study, however, we screened only the collected sera samples. We included negative sera as a reference.

### 4.4. Statistical Analysis

The continuous variables of the entire cohort were described as a mean (±standard deviation), whereas categorical variables were described as frequencies and percentages. Categorical variables of the different groups were compared through Fisher’s exact test. Statistical significance was set at a *p* value of < 0.05. Whenever the *p* value was between 0.05 and 0.09, even if non-statistically significant, it was reported as a number to highlight its trend. When the *p* value was >0.09, it was reported as “NS” (non-significant). Moreover, where necessary, a logistic regression analysis was performed to control the potential confounding effects of multiple predictor variables.

## 5. Conclusions

The world’s overcrowding and global warming highlight the need to find new edible resources as an alternative to traditional animal proteins; one example of an alternative is edible insects. Given their nutritional features and sustainability, further insect species are currently being considered by the EFSA for commercialization as human food, one of which is black soldier fly (*H. illucens*) larvae, largely employed in animal feeding. In light of the wide overlap of pan-allergens within the *Arthropoda* phylum, this observational study highlighted a higher reactivity of sera from patients sensitized to crustaceans and/or mites to recombinant _HI_TPM, compared to those from non-sensitized patients. Moreover, patients sensitized to both crustaceans and mites reported a significant higher rate of history of allergic reactions to food within the subset positive for IgE-reactivity to _HI_TPM, compared to _HI_TPM+ controls. However, knowledge about the effective allergenic risk represented by human ingestion of *H. illucens* is still in its infancy; therefore, targeted studies involving additional clinical tests, such as BAT, SPT and DBPCOFC, are needed, as well as investigations also involving other allergologically relevant arthropods, i.e., *A. simplex* and cockroaches.

## Figures and Tables

**Table 1:** Specific characteristics of the enrolled cohort.

	Study group (n 30)	Ctrl group (n 18)	*p*
Male gender	12 (40%)	9 (50%)	NS
Mean age ± sd (years)	41.6 ± 7.4	54.1 ± 12.2	<0.05
Sensitization only to HDM	11 (37%)	0	<0.05
Sensitization only to Crustaceans	0	0	NS
Sensitization to both HDM and crustaceans	19 (63%)	0	<0.05
Positive history of insects’ consumption	0	0	NS
Positive history of previous allergic reaction to food	10 (33%)	0	<0.05
	HDM+Cr− subset 0 (0)		NS
	HDM+Cr+ subset 10 (53%)		<0.001

**Table 2:** Rate of IgE-reactivity to _HI_TPM and _HI_AK in the STUDY and CTRL groups upon dot blot assays, and rate of history of allergic episodes in patients that were positive upon dot blot assays.

	Study group (n 30)	Ctrl group (n 18)	*p*
_HI_TPM+	22 (73%%)	8 (44%)	**0.045**
	HDM+Cr− subset 8 (73%)		NS
	HDM+Cr+ subset 14 (74%)		NS
_HI_AK+	23 (79%)	2 (40%)	NS
	HDM+Cr− subset 9 (82%)		NS
	HDM+Cr+ subset 14 (78%)		NS
History of food allergy in _HI_TPM+ subjects	7 (32%)	0 (0%)	NS
	HDM+Cr− subset 0 (0%)		NS
	HDM+Cr+ subset 7 (50%)		**0.022**
History of food allergy in _HI_AK+ subjects	7 (30%)		NS
	HDM+Cr− subset 0 (0%)	0 (0%)	NS
	HDM+Cr+ subset 7 (50%)		NS

## Data Availability

The data that support the findings of this study are available from the corresponding author upon reasonable request.

## Abbreviations

The following abbreviations are used in this manuscript:

_HI_TPM	tropomyosin of *Hermetia illucens*
_HI_AK	arginine kinase of *Hermetia illucens*
Cr	crustaceans
HDM	house dust mites
EFSA	European Food Safety Authority
TPM	tropomyosin
AK	arginine kinase
BAT	basophil activation test
SPTs	skin prick tests
DBPCOFC	double-blind placebo-controlled oral food challenge test
BSA	bovine serum albumin
